# Male grower pigs fed cereal soluble dietary fibres display biphasic glucose response and delayed glycaemic response after an oral glucose tolerance test

**DOI:** 10.1371/journal.pone.0193137

**Published:** 2018-03-01

**Authors:** Anton M. Pluschke, Barbara A. Williams, Dagong Zhang, Stephen T. Anderson, Eugeni Roura, Michael J. Gidley

**Affiliations:** 1 Queensland Alliance for Agriculture and Food Innovation, Centre for Nutrition and Food Sciences, The University of Queensland, St Lucia Brisbane, Australia; 2 School of Biomedical Science, The University of Queensland, St Lucia Brisbane, Australia; INIA, SPAIN

## Abstract

Acute and sustained soluble dietary fibre (SDF) consumption are both associated with improved glucose tolerance in humans and animal models (e.g. porcine). However, the effects on glucose tolerance in grower pigs, adapted to diets with a combination of SDF have not been studied previously. In this experiment, cereal SDF wheat arabinoxylan (AX) and oat β-glucan (BG) were fed individually and in combination to determine the effect on glucose tolerance in jugular vein catheterized grower pigs. Five groups of Large White male grower pigs were fed highly digestible diets containing either 10% AX, 10% BG, 5% AX with 5% BG, a model cereal whole wheat flour (WWF), or a control wheat starch diet (WS) with no SDF. Blood was collected via jugular vein catheters over 240 minutes following a feed challenge and an oral glucose tolerance test (OGTT) on two separate days. Postprandial blood samples were used to determine plasma glucose, insulin, non-esterified fatty acids (NEFA), glucose-dependent insulinotropic polypeptide (GIP), glucagon-like peptide-1 (GLP-1), peptide tyrosine tyrosine (PYY), ghrelin, glucagon and cortisol concentrations. No dietary effects on glycaemic response were observed following the feed challenge or the OGTT as determined by the area under the curve (AUC). A biphasic glucose and insulin response was detected for all pigs following the OGTT. The current study showed male grower pigs have tight glycaemic control and glucose tolerance regardless of diet. In addition, pigs fed the combined SDF had a reduced GIP response and delayed insulin peak following the feed challenge. Incretin (GLP-1 and GIP) secretion appeared asynchronous reflecting their different enteroendocrine cell locations and response to nutrient absorption.

## Introduction

Soluble dietary fibres (SDFs) have been reported to have numerous health benefits for humans, including reducing postprandial blood glucose levels, and improving both overall glycaemic response [1, 2] and insulin sensitivity [3]. SDFs are expected to form viscous solutions, aggregates or gels when they are exposed to an aqueous liquid phase [4]. In forming these structured systems, SDFs have been observed to increase gastric retention time primarily due to increased viscosity of gastric contents [5, 6]. Human and animal studies have shown that the consumption of SDF delays gastric emptying and reduces the rate of digestion and absorption of macronutrients in the small intestine [7, 8]. These actions are seen as beneficial to human health as they result in lower postprandial glycaemic and insulin responses [9–12]. Pigs have been recently reaffirmed as being an appropriate model for human nutritional physiology [13] and suitable as models for children and adolescents in diabetes research [14].

Intact whole cereal grains have proved to be effective in reducing glucose and insulin responses [15]. While whole cereal grains consist of a combination of SDF and insoluble dietary fibre (IDF), the ratio of SDF to IDF depends on the cereal grain [16]. Few studies combine more than one type of SDF in an experimental diet. Typically, the amount or type of SDF is varied which allows potential diet effects to be explored. Cereals are a major source of fibre in the human diet, with two main SDF components, arabinoxylan (AX) and mixed-linkage β-glucan (BG). The current study included diets with these two types of SDF, alone or in combination, compared with whole wheat flour and a diet without SDF.

Although the link between enteroendocrine feedback regulating postprandial insulin secretion and glycaemia has been studied in human and animal models, little is known about the influence of SDF. The incretin hormones glucose-dependent insulinotropic polypeptide (GIP) and glucagon-like peptide-1 (GLP-1) are known to be potent determinants of the postprandial insulin release that occurs after increases in blood glucose [17, 18]. Additionally, GLP-1 has also been shown to enhance satiety and reduce food intake in humans and animal models [19]. Likewise, additional effects of GIP include enhanced energy storage via direct actions on adipose tissue including the release of lipoprotein lipase from fat, as well as increasing the rate of lipogenesis and triglyceride synthesis. Moreover, fat cells are known to have GIP receptors and GIP has been shown to increase the clearance rate of chylomicrons in the circulation and can inhibit the action of glucagon [20].

This study aims to determine the effect of SDF AX and BG (individually and in combination, i.e. AXBG) on the glycaemic response in grower pigs following an oral glucose tolerance test (OGTT) and a feed challenge. We hypothesized that adding SDF to the diet would improve the glycaemic response (i.e. lower postprandial blood glucose and insulin response). It was further hypothesized, that pigs fed SDF in combination would present intermediate glycaemic responses compared with the individual SDFs.

## Materials and methods

A complete randomized block design was used and the Animal Ethics Committees of the University of Queensland approved all animal experimental procedures (Animal Ethics Committee Approval Number: NFS/364/13/ARC).

### Animals and housing

Thirty Large White male pigs (boars) were housed in a temperature controlled room (22°C ± 2°C), at the University of Queensland (Gatton Campus Piggery). Pigs were introduced to their experimental diet over a seven day period with six pigs per diet. Pigs were housed individually on raised floors in 1.8 m^2^ pens. The walls of the pens were covered in plastic resulting in smooth walls. Individual housing was realised by placing stainless steel pig pens side-by-side with a feeding trough at one end. Pigs could see and hear each other but were restricted by the pens to touch. Lights were on from 0600 to 1800 h and the shed was mechanically ventilated.

### Experimental design

The experiment was split into three groups over a consecutive three-week period, with 10 pigs per period. Pigs weighed 48 ± 4 kg at the beginning of the experiment. Four experimental diets (and one control diet) were fed twice daily in each period (two pigs per diet, per period), and the pigs had *ad libitum* access to water. Blood samples were collected after the pigs had been fed for two weeks on the diet.

### Ingredients, diets and feeding

Oat BG was obtained from Garuda International, Ca. USA, while the wheat arabinoxylan-rich fraction was obtained from a gluten extraction plant and analysed to contain 39% arabinoxylan with most of the rest of the fraction consisting of starch. Whole wheat flour was obtained from local sources. The ingredients of the diets (Table 1) were based on wheat starch, Na-caseinate, whey protein concentrate (80%), whole egg powder, sucrose, palm oil, and sunflower oil with added vitamins and minerals, which were also sourced locally. The SDF replaced wheat starch on an energy and percentage basis. The four experimental diets were all balanced on an energy basis (Table 2). The control contained wheat starch (WS) as the primary source of carbohydrate, the AX diet contained wheat arabinoxylan at 10% inclusion, the BG diet contained oat mixed linkage β-glucan at 10% inclusion, the combination diet contained AX and BG at 5% inclusion each. The whole wheat flour (WWF) diet was balanced with other diets in terms of nutrient and energy profile. Pigs were fed individually (twice a day at 0800 and 1600 hrs) according to their metabolic weight at 2.7 times the metabolisable energy requirements for maintenance. Feeding times were varied in the morning so the pigs would not associate the first person to enter the room with the one who feeds them. Daily feed allocation increased weekly, based on body weight.

**Table 1:** Composition of experimental diets (g.kg^-1^) based on wheat starch and fed to the pigs for a minimum of 14 days.

Diet Ingredients	WS	AX	BG	AXBG	WWF
g/kg DM					
Whole wheat flour	0	0	0	0	589.1
Soluble beta glucan	0	0	117.3	58.7	0
Soluble arabinoxylans^1^	0	255.2	0	127.6	0
Wheat starch	429.1	193.9	306.8	250.8	0
Na-caseinate	50	50	50	50	50
Whey protein concentrate 80%	100	80	105	92	0
Whole egg powder	150	150	150	150	150
Sucrose	50	50	50	50	50
Cellulose (Arbocel RC fine)	60	60	60	60	0
Palm oil	60	60	60	60	60
Sunflower oil	40	40	40	40	40
Vitamin trace element mix*	2	2	2	2	2
Celite	20	20	20	20	20
CrCl3	0.9	0.9	0.9	0.9	0.9

*Vitamin and mineral mix provided Limestone 15, Dicalcium phosphate 13, NaHCO3 6, Salt (NaCl) 3, MgO 1 (g.kg^-1^) to all diets

^1^arabinoxylan fraction from wheat extraction containing primarily AX (39%) and starch.

Abbreviations: WS, Control diet; AX, 10%arabinoxylan diet; BG, 10% beta-glucan diet; AXBG, 5%AX and 5%BG diet; WWF, Whole wheat flour diet

**Table 2:** Calculated nutritional composition (g.kg^-1^) of the experimental diets.

Nutrient composition	WS	AX	BG	AXBG	WWF
g/kg DM					
DM	902.5	916.6	912.2	914.4	886.2
DE (MJ/Kg)	17.4	16.9	17.1	17.0	16.6
Crude ash	45.6	50.8	48.7	49.7	50.1
Crude protein	197.0	200.9	200.3	200.2	201.3
Dig crude protein	178.2	181.0	181.2	180.7	175.1
Crude fat	170.6	169.4	170.9	170.1	170.2
Starch	373.7	284.0	270.0	277.4	352.3
Sol NSP	0.0	96.0	106.4	101.2	5.6
Insoluble NSP	53.3	55.1	53.3	54.2	53.3
AX total	0.0	91.6	2.3	47.0	23.6
BG total	0.0	4.3	91.2	47.8	5.1
AX soluble	0.0	91.6	2.3	47.0	3.9
AX insoluble	0.0	0.0	0.0	0.0	19.6
BG soluble	0.0	4.3	91.2	47.8	1.7
BG insoluble	0.0	0.0	0.0	0.0	3.5

Abbreviations: DM, Dry Matter; DE, Dietary Energy; WS, Control diet; AX, 10%arabinoxylan diet; BG, 10% beta-glucan diet; AXBG, 5%AX and 5%BG diet; WWF, Whole wheat flour diet

After an overnight fast, pigs were given the feed challenge (i.e. half daily feed allowance offered at once) and the time taken to finish the meal was recorded. All pigs consumed their meal within 10 ± 5 minutes. The following day the same pigs received an OGTT. After an overnight fast, a glucose solution (1.5g/kg body weight, glucose dissolved in deionized water was fed orally to the pigs in large syringes. Silicon tubes were attached to the ends of the 50 mL syringes, and was fed to them by the operators, allowing the pigs to drink the glucose solution with minimal loss. All pigs consumed the glucose solution within 2 ± 1 minutes. Blood was sampled immediately after both the feed challenge and OGTT.

### Habituation

Prior to surgery and blood sampling, pigs were handled on a daily basis by a number of trained personnel. Each pig experienced an extensive amount of human contact, with a particular focus on the region of the neck and ears so as to habituate them to human contact and allow for effective stress-free blood sampling via the jugular catheters. Pigs were also trained to drink the glucose solution via the silicon extension tubes.

### Surgery

A detailed account of the surgical proceedings has been reported [21]. In brief, the pigs (60 ± 2 kg) were sedated, anesthetized and placed dorsally on an operating table. Each pig received an intramuscular injection of 0.15 mg/kg of butorphanol (Butorgesic®, Ilium Veterinary Products, Australia) and 3 mg/kg of Zoletil® (Virbac Animal Health, Australia). They were then delivered isoflurane (IsoFlo®, Abbott laboratories, Australia) in oxygen by mask while a 20 G catheter was secured in an ear vein. Some pigs were sufficiently anaesthetised without further drug administration, but most required a small dose of thiopentione sodium IV (2.5 mg/kg, 0–4.8 mg/kg) (Ilium Thiopentone®, Troy Laboratories, Australia). Following intubation, an incision was made in the jugular fossa on one side of the neck in a caudo-medial to cranio-lateral direction. Blunt dissection was used and once the jugular vein was located, a central venous catheter was inserted into the vein (Central Venous catheterization set with Blue Flextip Catheter, Arrow Deutschland GmbH, Erding, Germany). The end of the catheter was then sutured in place adjacent to the jugular vein with 3/0 chromic catgut. Subcutaneous tissues were also sutured with 3/0 catgut in order to minimise dead-space. An intravenous extension tube was secured onto the end of the catheter at the cranial end of the skin incision and the wound was closed with surgical staples and a sterile dressing placed over the wound. The intravenous extension tube was secured by means of tape and Superglue (Selleys Pty Ltd, Padstow, NSW, Australia) onto the back of the neck. Following the operation, pigs were given two days to recover before the feed challenge. Catheters were flushed with heparinised saline (4IU) to maintain patency following each blood removal and a heparin lock of 100IU was administered during the evening.

### Sampling protocol

Blood was collected in heparinised Vacutainers^TM^ for insulin analysis and in Vacutainers^TM^ coated with anti-coagulant K_2_EDTA and a peptidase inhibitor cocktail containing Protease, Esterase and DPP-IV Inhibitors (BD^TM^ P800 366421). Blood was collected one hour prior to feeding (-60 min) and then every 15 minutes from feeding time (T = 0 min) to two hours (120 min) postprandial and then at three (180 min) and four hours (240 min) postprandial. Following each blood collection (approx. 25 ml), catheters were flushed with 10–20 mL of 0.9% saline solution to replace fluid loss followed by 3 mL of heparinised saline (4IU) to prevent clotting. Following the 240 min blood collection and saline flushing, a 3 mL heparin lock (100IU) was issued to maintain patency. The volume of heparinised saline was 3 mL, which was the volume of the catheter and the two extension tubes. Blood was centrifuged at 3000 g for ten minutes and plasma frozen at -20°C and stored at -80°C for peptide hormone analysis.

### Analytical procedure

Plasma samples were analysed for glucose according to a standard procedure using glucose hexokinase II and enzymatic colorimetric determination. Non-esterified fatty acid (NEFA) was determined using the Wako ACS-ACOD assay using a Beckman Coulter AU400 Analyser (Beckman Coulter Inc. Brea California USA) and within and between assay precision of 3.0%.

Plasma insulin was analysed by RIA (Siemens Coat-a-Count kit 10381347), with assay sensitivity of 0.9 mIU/mL, and within and between assay precision of 6.7% and 7.9% respectively for a quality control of 22.9 mIU/mL. Cortisol was analysed by RIA (Beckman Coulter kit IM1841) with assay sensitivity of 2.5 nM, and within assay precision of 2.7% for a quality control of 139.9 nM. Glucagon was analysed by RIA (Millipore Glucagon kit GL-32K) with assay sensitivity of 12.5 pg/mL, and within and between assay precision of 3.0% and 3.0% respectively for a quality control of 110.7 pg/mL. GLP-1 was analysed by RIA (Millipore GLP-1 active kit GLP1A-35HK) with assay sensitivity of 3pM, and within and between assay precision of 5.2% and 3.7% respectively for a quality control of 40.0 pM. GIP was analysed by ELISA (Millipore GIP kit EZRMGIP-55HK) with assay sensitivity of 8.2 pg/mL, and within and between assay precision of 0.7% and 0.4% respectively for a quality control of 540.7 pg/mL. Peptide YY was analysed by RIA (Millipore kit PCP-22K) with assay sensitivity of 0.1 ng/mL, and within and between assay precision of 4.2% and 5.8% respectively for a quality control of 188.4 pg/mL. Ghrelin was quantified using a commercial RIA kit (GHRT-89HK; Total Ghrelin; EMD Millipore). Plasma glucose, insulin and NEFA concentrations were determined for all time points (-60 min to 240 min). Plasma cortisol concentrations were determined for all pigs at time points 0 and 120 min. Plasma GIP, GLP-1, PYY and ghrelin concentrations were determined for pigs fed control (WS) and AXBG diets following the feed challenge.

### Statistical analyses

All statistical analyses were performed using SAS 9.3 (SAS Institute, Inc., Cary, NC, USA). GraphPad Prism (Ver. 6.04) was used to compute the AUC by the trapezoidal method. The AUC was determined for overall (i.e. 0 to 240 min) and for 30 minute time intervals (i.e. 0 to 30, 30 to 60, 60 to 90 min etc.). In addition, plasma peak concentrations were determined between diets. The AUC for plasma glucose, insulin, NEFA and gut peptide concentrations were analysed on diet, time and diet*time effects using the MIXED procedure, using diet as a repeated measure, with pig as subject. After an interaction was found, diet effects per time were analysed using the slice statement in the MIXED procedure. If the slice statement reported significant differences, a Post Hoc test using the Tukey method was further used to determine differences between groups. Group means were analysed using the GLM procedure and significance of treatment differences was set at *P*<0.05. Tendencies were reported when 0.05<*P*<0.1.

## Results

The pigs appeared healthy and consumed their daily feed allowances for the entire experiment. The surgery had no complications [21].

### OGTT

**Fig 1:**
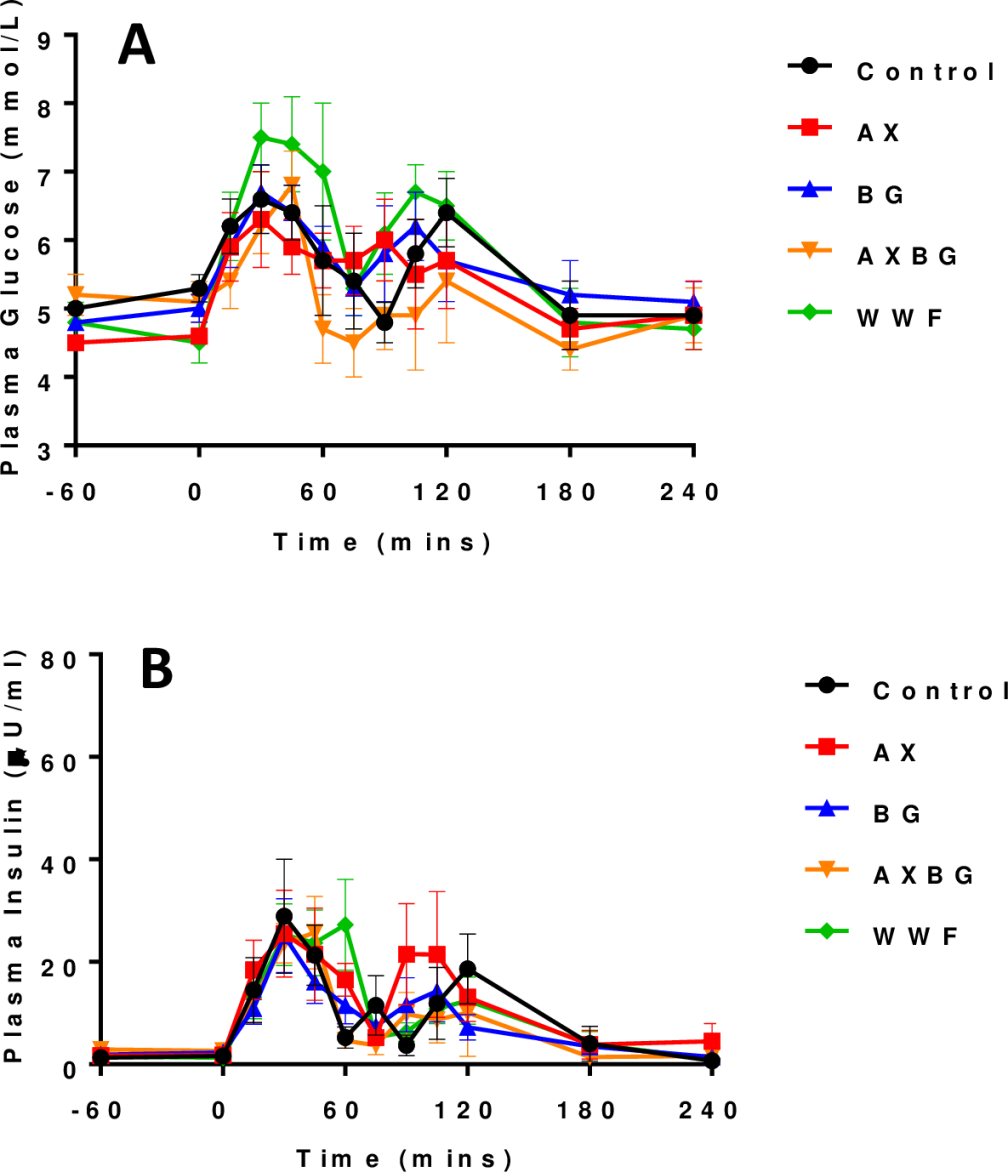
Plasma glucose (A) and insulin (B) following the OGTT.

Both the glycaemic and insulin responses following the OGTT were biphasic (Fig 1A and 1B respectively). Diet had no significant effect on plasma glucose concentration (*P* = 0.256) and insulin (*P* = 0.524) responses, following the OGTT in terms of AUC, neither overall (i.e. 0 to 240 min) nor when analysed in 30 minute segments (i.e. 0 to 30, 30 to 60, 60 to 90 etc.). The glycaemic response resulting from the OGTT was not significantly different (AUC) when compared with the AUC from the feed challenge (*P* = 0.151); however, the profiles between each challenge were markedly different. The insulin response following the OGTT was significantly smaller (AUC) than the insulin response following the feed challenge (*P*<0.001).

Following the OGTT, plasma glucose and insulin AUC from -60 (i.e. 1 h before feeding) to 240 minutes postprandial were determined and the results presented in Table 3. The insulin response following the feed challenge was significantly larger (*P*<0.001) when compared with the OGTT.

**Table 3:** Plasma glucose (mmol.L^-1^/min) and insulin (μU.ml^-1^/min) area under the curve (AUC) from the oral glucose tolerance test of pigs adapted to and fed different diets.

AUC		WS	SE	AX	SE	BG	SE	AXBG	SE	WWF	SE	*P-value*
	n	6		6		7		5		6		Diet
OGTT Glucose		1642.0	26.3	1560.7	61.9	1642.9	68.5	1521.4	90.7	1685.2	33.8	NS
OGTT Insulin		2512.7	424.9	2924.3	430.7	2116.6	260.4	2026.6	403.5	2504.1	553.8	NS

Where OGTT = Oral glucose tolerance test and WS = control diet, AX = arabinoxylan diet, BG = β-glucan diet, AXBG = combination diet, WWF = whole wheat flour diet, NS (not significant)

Following the OGTT, blood glucose levels responded in a biphasic manner. For the first phase, they increased to between 6.3 ± 0.7 and 7.5 ± 0.5 mmol/L and peaked at 30 minutes except for pigs fed the AXBG diet which peaked at 45 minutes (Table 4). For the second phase, blood glucose levels increased to between 5.4 ± 0.9 and 6.7 ± 0.4 mmol/L and this occurred between 90 and 120 min postprandially depending on the diet. Plasma insulin levels peaked initially between 25.1 ± 7.2 and 28.9 ± 11.1 µU/ml and occurred primarily at 30 minutes postprandial, except for pigs fed the AXBG and WWF diets, which occurred at 45 and 60 minutes respectively. Following a trough after the first phase, plasma insulin levels increased, again peaking between 10.1 ± 8.5 and 21.5 ± 9.9 μU/ml occurring predominately at 120 minutes postprandial.

**Table 4:** Glucose (mmol/L) and insulin (μU/ml) peaks following the OGTT.

OGTT	Glucose peak #1—Time	Insulin peak #1—Time	Glucose peak #2—Time	Insulin peak #2—Time
WS	6.6±0.5–30 min	28.9±11.1–30 min	6.4±0.5–120 min	18.6±6.8–120 min
AX	6.3±0.7–30 min	25.5±8.5–30 min	5.7±0.7–120 min	21.5±9.9–90 min
BG	6.7±0.4–30 min	25.1±7.2–30 min	6.0±0.6–90 min	14.3±6.0–105 min
AXBG	6.8± 0.5–45 min	25.7±7.1–45 min	5.4±0.9–120 min	10.1±8.5–120 min
WWF	7.5±0.5–30 min	27.2±8.9–60 min	6.7±0.4–105 min	12.4±4.6–120 min

Where Time represents the time (min) when the peak occurred following the OGTT

### Feed challenge

**Fig 2:**
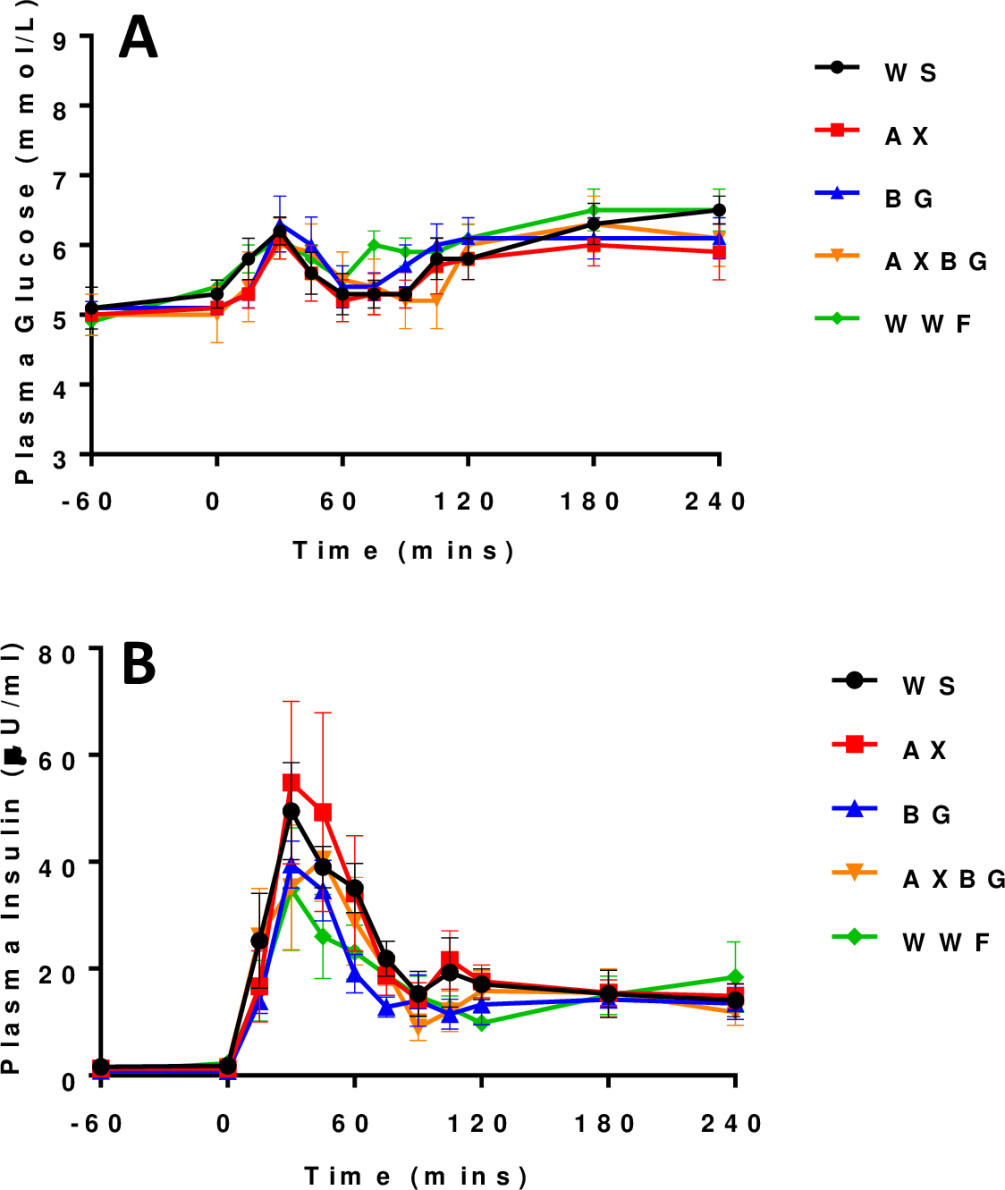
Plasma glucose (A) and insulin (B) following the feed challenge.

No dietary effect was observed (*P* = 0.595) on the AUC for plasma glucose from -60 to 240 minutes postprandial following the feed challenge (Table 5 & Fig 2A). During the feed challenge, a second sustained rise of plasma glucose from 120 to 240 minutes postprandial was observed, and in the case of WWF pigs was greater (but not significantly different—*P*>0.05) than the first glucose peak (6.1±0.3 mmol/L at 30 min and 6.5±0.3 mmol/L at 180 min). No dietary effect was observed (*P* = 0.347) for plasma insulin on the AUC from -60 to 240 minutes postprandial (Fig 2B). In the course of the feed challenge, plasma insulin concentrations rose rapidly, peaking at 30–45 min and did not return to fasting/basal levels over the four hours of sampling (S1 File).

**Table 5:** Plasma glucose (mmol.L^-1^/min) and insulin (μU.ml^-1^/min) area under the curve (AUC) from the feed challenge of pigs adapted to and fed different diets.

AUC		WS	SE	AX	SE	BG	SE	AXBG	SE	WWF	SE	*P-value*
	n	6		6		7		5		6		Diet
Feed Glucose		1730.2	56.2	1669.5	72.4	1727.0	60.6	1700.0	92.2	1788.6	45.5	NS
Feed Insulin		5172.7	485.3	5267.3	1130.2	3990.3	198.7	4536.2	678.2	4119.3	708.1	NS

Where Feed = Feed challenge and WS = control diet, AX = arabinoxylan diet, BG = β-glucan diet, AXBG = combination diet, WWF = whole wheat flour diet, NS (not significant)

Fasting blood glucose levels were 5 mmol/L and increased to 6.1 mmol/L following the feed challenge. This increase (amount and time taken) in plasma glucose concentrations was consistent for all pigs (Fig 2A). Regardless of diet, pigs reached peak plasma glucose concentrations at 30 minutes postprandial (Table 6). On average, fasting insulin levels were 1.1 μU/ml and peaked at 43.8 μU/ml for all pigs at 30 minutes following the feed challenge. However, pigs fed the AXBG diet experienced a delayed insulin peak that occurred at 45 min.

**Table 6:** Glucose and insulin peaks following the feed challenge (±SE).

Feed	Glucose peak—Time	Insulin peak—Time
WS	6.2±0.2–30 min	49.5±9.1–30 min
AX	6.1±0.3–30 min	54.8±15.2–30 min
BG	6.3±0.4–30 min	39.5±4.4–30 min
AXBG	6.0±0.2–30 min	40.4±17.5–45 min
WWF	6.1±0.3–30 min	34.9±11.5–30 min

**Fig 3:**
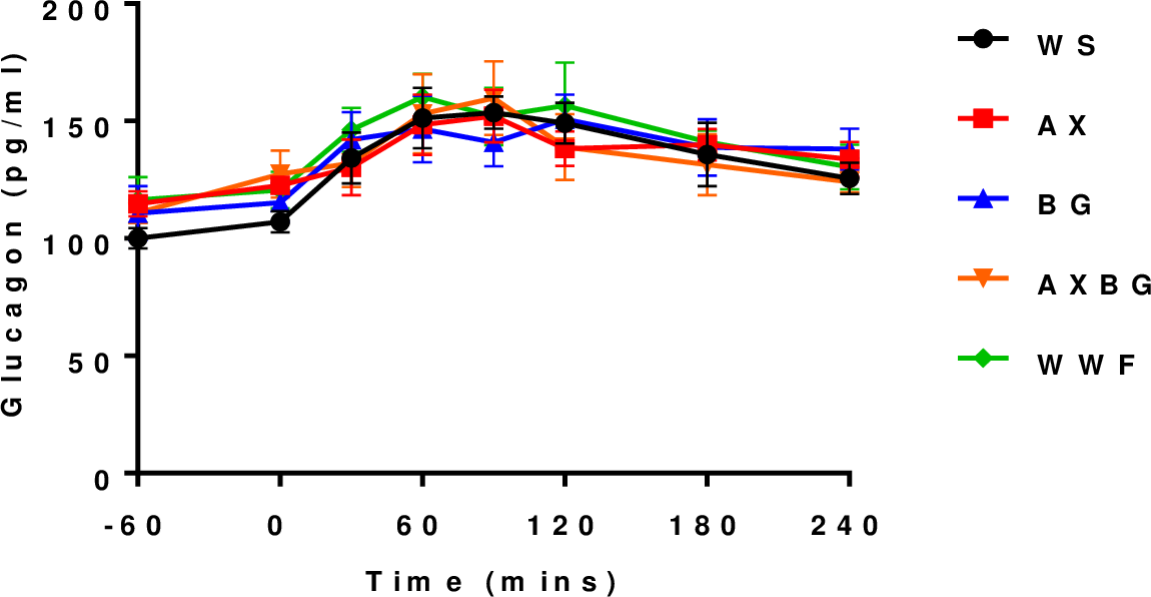
Plasma glucagon concentration following a feed challenge.

To further elucidate the sustained rise of plasma glucose following the feed challenge, plasma glucagon concentrations were determined at times -60, 0, 30, 60, 90, 120, 180 and 240 minutes in all pigs (Fig 3); No apparent diet effects (AUC) were observed for plasma glucagon following the feed challenge (*P* = 0.164). Plasma glucagon concentrations increased over 240 minutes postprandial. Plasma glucagon concentrations were highest between 60–120 minutes postprandial (but not significantly different from plasma glucagon concentrations at time 0, *P* = 0.430), corresponding with declining plasma glucose and insulin concentrations. However, after the feed challenge, plasma glucagon concentrations were increased throughout the sampling period while at 240 minutes postprandial all pigs had continued higher plasma glucose, insulin and glucagon concentrations.

### NEFA–OGTT and feed challenge

**Fig 4:**
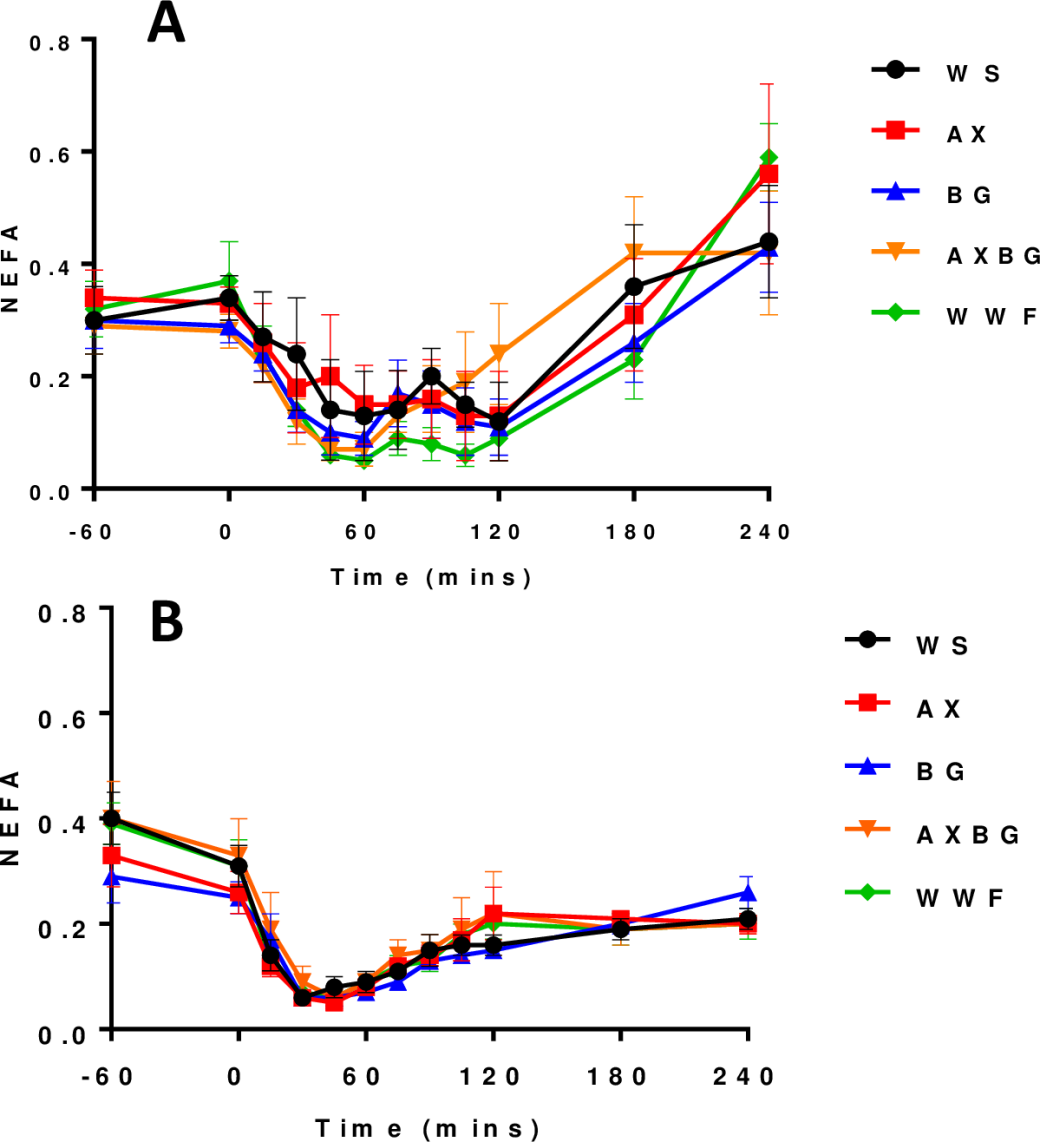
Plasma non-esterified fatty acids from the OGTT (A) and the feed challenge (B).

Plasma NEFA concentrations (AUC) were not subject to dietary effects from -60 to 240 minutes postprandial following the OGTT or the feed challenge (P = 0.94 and 0.85 respectively) (Fig 4A and 4B). Plasma NEFA concentrations plateaued after 120 minutes following the feed challenge; however, they continued to increase after 120 minutes following the OGTT.

### Incretins (GIP & GLP-1), PYY and ghrelin responses

**Fig 5:**
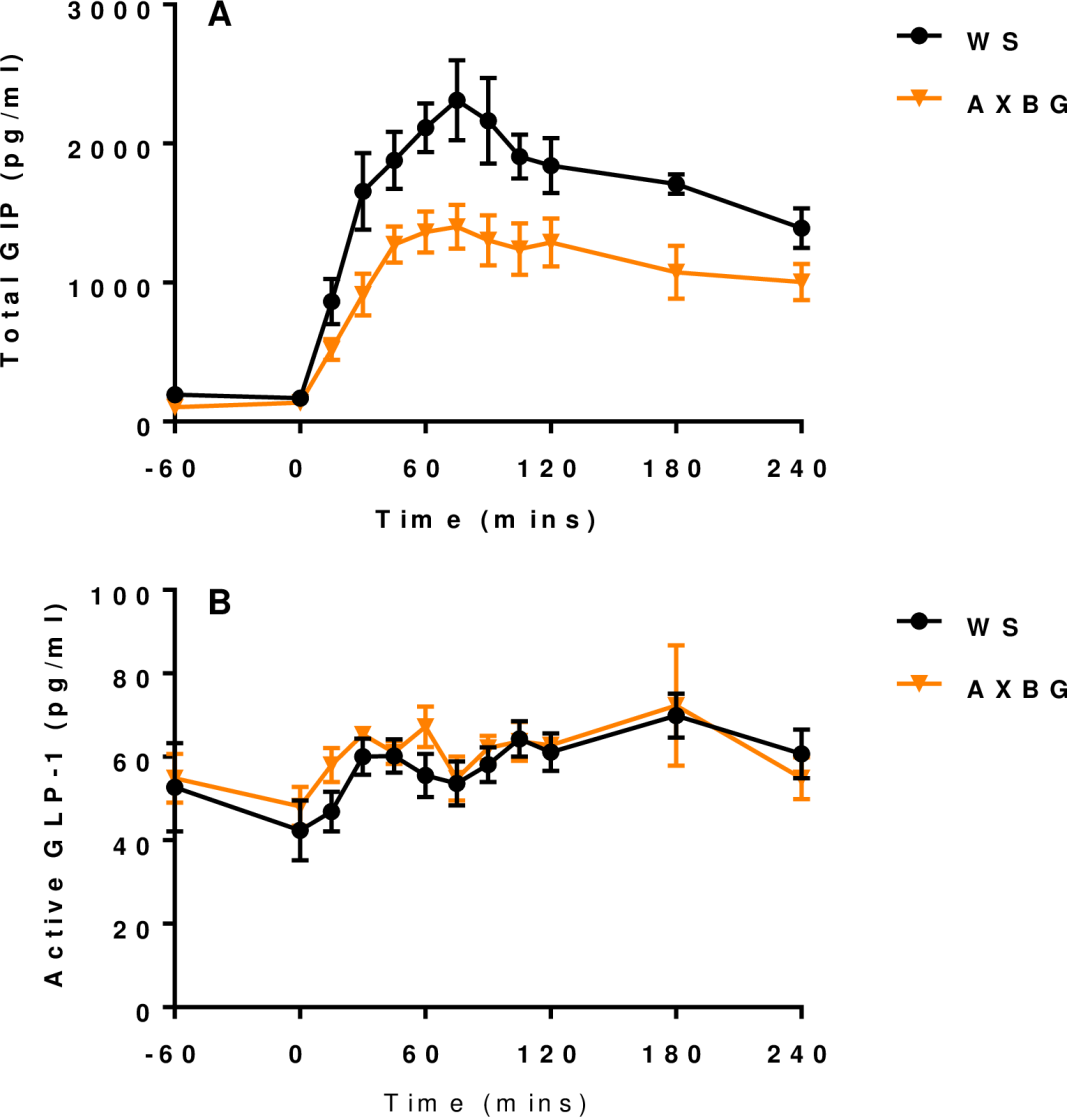
Total plasma GIP (A) and active plasma GLP-1 (B) concentrations following a feed challenge of pigs adapted to and fed different diets where WS = control diet and AXBG = combination diet.

Given that all diets showed similar results except for AXBG, it was decided to compare the plasma incretin concentrations (GIP and GLP-1) for pigs fed the WS and AXBG diets. Postprandial responses to the feed challenge are presented in Fig 5. Plasma GIP concentrations (AUC) were significantly lower in pigs fed the AXBG diet when compared with pigs fed the WS diet (*P* = 0.002) from -60 to 240 minutes postprandial (Fig 5A). Plasma GLP-1 concentrations increased over time, reaching a maximum at 180 min for pigs fed both diets, however this peak was not significantly higher (*P* = 0.246) than the basal level (i.e. time 0) (Fig 5B). Pigs fed AXBG had a numerically but not significantly higher plasma GLP-1 AUC (*P* = 0.725) when compared with pigs fed the WS diet.

**Fig 6:**
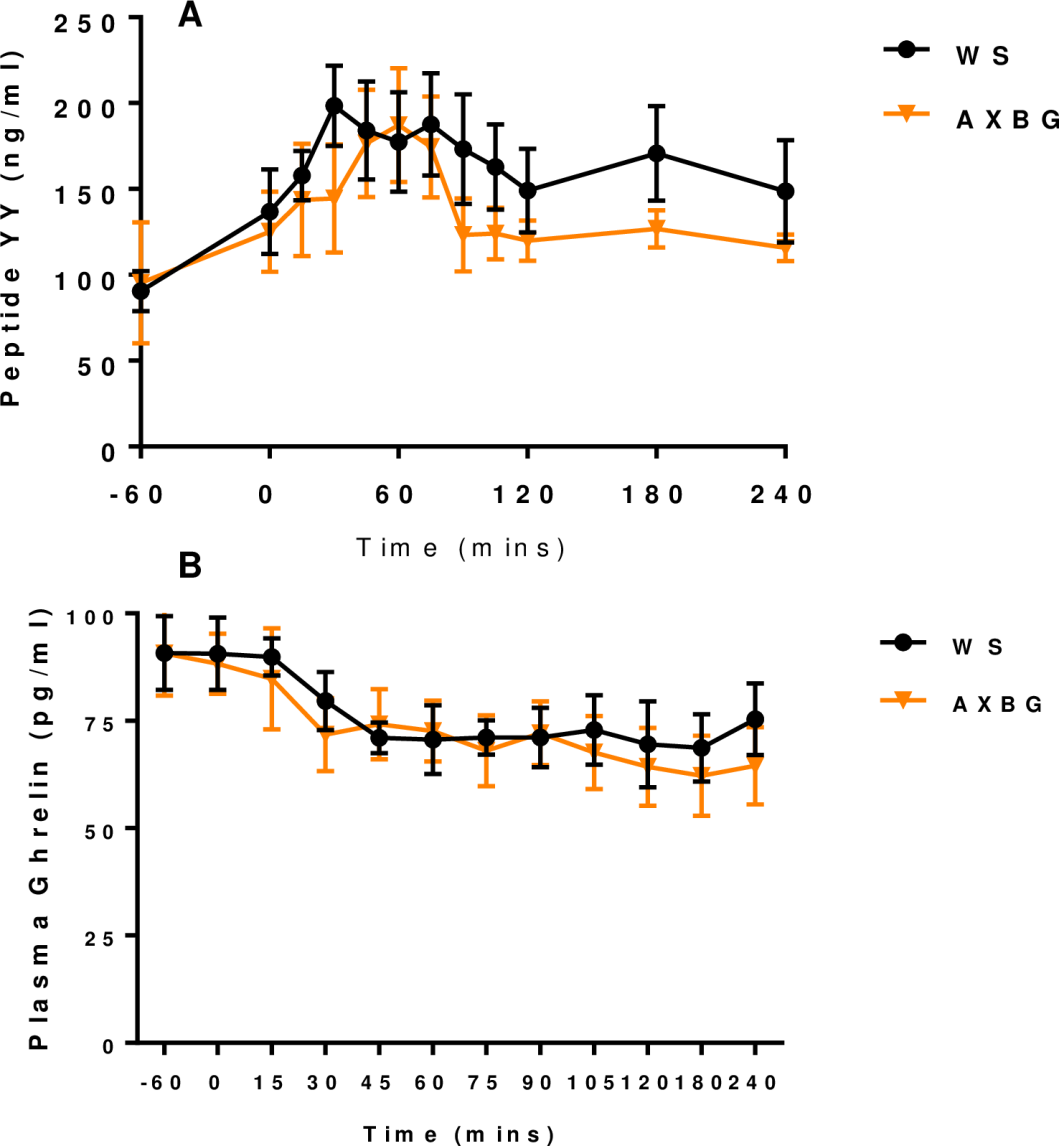
Plasma PYY (A) and ghrelin (B) concentrations following a feed challenge of pigs adapted to and fed different diets where WS = control diet and AXBG = combination diet.

Plasma PYY and ghrelin concentrations were determined for pigs fed WS and AXBG diets following the feed challenge and are presented in Fig 6A and 6B respectively. There was no difference in fasting levels of PYY determined at -60 minutes (*P* = 0.485). Prior to feeding, plasma PYY concentrations increased from 100 to 140 ng/ml, although this increase was not significant (*P* = 0.751). PYY levels continued to increase in response to feeding and peaked at 30 minutes for pigs fed the WS diet. Pigs fed the AXBG diet, peaked at 60 minutes postprandial. Overall, plasma PYY levels (AUC) were not significantly lower in pigs fed the AXBG diet compared with pigs fed the WS diet (*P* = 0.266). After both peaks, plasma PYY levels returned to fasting levels, however this occurred more rapidly (i.e. after 30 minutes) for pigs fed the AXBG diet compared with after 90 minutes for pigs fed the WS diet. Plasma ghrelin levels decreased during the feed challenge for pigs fed both WS and AXBG diets. Plasma ghrelin (AUC) levels were numerically but not significantly lower (*P* = 0.603) for pigs fed the AXBG diet as compared with pigs fed the WS diet (S2 File).

### Cortisol response

Following the feed challenge, plasma cortisol concentrations were determined at time 0 and 120 minutes postprandial (Table 7). Although eight pigs had plasma cortisol concentrations that were raised (~50 ng/ml), no plasma cortisol concentrations were sufficiently elevated (i.e. > 70 ng/ml) to indicate severe stress [22].

**Table 7:** Plasma cortisol (ng/mL) (mean ± SE) at times 0 and 120 mins postprandial of pigs adapted to and fed different diets where WS = control diet, AX = arabinoxylan diet, BG = β-glucan diet, AXBG = combination diet, WWF = whole wheat flour diet.

Time (mins)		WS	SE	AX	SE	BG	SE	AXBG	SE	WWF	SE	*P-value*	
	n	6		6		7		5		6		Diet	Time
0		31.1	6.2	26.1	7.3	36.5	7.7	29.3	5.7	45.4	11.5	NS	**
120		13.2	4.7	7.9	1.4	29.4	13.6	22.1	9.1	22.5	9.6		

NS (not significant) and

** *P* <0.01

## Discussion

Intake of SDF is expected to lower glucose and insulin responses.

The current study aimed to determine the effect of SDFs AX and BG (individually and in combination) on the glycaemic response in grower pigs following an OGTT and a feed challenge. Pigs fed cereal SDFs for a period of two weeks did not demonstrate a reduced glucose or insulin response following either an OGTT or a feed challenge. In addition, as detailed in the following sections, the study revealed the following:

1. A biphasic postprandial glucose response was observed following the OGTT for all pigs which therefore predicts good glucose tolerance.
2. Diet (i.e. SDF) did not affect the glycaemic response (AUC) following either the feed challenge or the OGTT due to apparent tight glycaemic control in all pigs.
3. Pigs fed a combination of SDF (i.e. AXBG) had significantly decreased postprandial plasma GIP but no difference in GLP-1, PYY or ghrelin responses.
4. SDF inclusion may account for differences observed in the incretin response due to reduced nutrient absorption occurring in the proximal duodenum.

### A biphasic postprandial glucose response was observed following the OGTT for all pigs and predicts good glucose tolerance

A biphasic plasma glucose response is more frequently reported in females (humans and pigs) and is therefore thought to be predominantly a female characteristic [23]. The results from the current study suggest otherwise, as these pigs were male and showed a biphasic response curve following the OGTT. The experimental design is crucial to being able to determine a biphasic response to an OGTT. In the current study, blood samples were collected over a four-hour period postprandial. The second peak in plasma glucose concentrations was observed at 120 min postprandial. Coincidently, 120 min is the second time point (other than time zero) when blood is taken from humans to detect their glycaemic response to an OGTT. This poses a potential issue as a biphasic response could in fact be eroding ‘true’ glucose tolerance in human studies. It is therefore recommended that when determining glucose tolerance in humans, blood be sampled frequently (i.e. at least every 15 minutes) after being issued an OGTT.

The rise in plasma glucose concentrations from 60 to 120 minutes (phase 2) is potentially due to the interplay between insulin and glucagon. As plasma insulin concentrations decreased between 30 and 60 minutes following the feed challenge, glucagon secretion increased ensuring that plasma glucose levels would remain within the homeostatic range. The glucoregulatory hormones of the body are designed to maintain circulating glucose concentrations and are constantly interacting in animals with tight glycaemic control [24]. These results support the notion of tight glycaemic control in these pigs. The continued elevated plasma glucose concentration (Fig 2A) is most likely a result of continued digestion and absorption occurring in the small intestine, prolonging the insulin response with some opposing glucagon action [25]. Increased glucagon concentrations would promote hepatic gluconeogenesis and hepatic glycogenolysis, however insulin would oppose this [26].

### Diet (i.e. SDF) did not affect the glycaemic response (AUC) following the feed challenge or the OGTT

Following the feed challenge, plasma glucose concentrations could be separated into three phases. The first phase from 0 to 60 minutes displayed a plasma glucose peak (30 min) and decline, which was followed by a second phase rise from 60 to 120 minutes, which then plateaued into a third phase from 120 to 240 minutes. As such, plasma glucose concentrations did not return to a resting state (~5 mmol/L) instead remaining at 6–6.5 mmol/L. The sustained concentration of plasma glucose from 120 to 240 minutes was thought to be due to continued digestion and absorption of the feed. As gastric retention time can typically last for over two hours (depending on the characteristics of the feed ingested), this plateau suggests that there is continued secretion of chyme through the pyloric sphincter into the small intestine. Plasma cortisol concentrations were not elevated sufficiently to cause excessive glucose production within liver cells (glycogenolysis) which has been shown to result in acute hyperglycaemia [27, 28].

A study by Hooda et al (2010) found a decreased glucose absorption in portal-vein catheterized pigs within the first hour after feeding with a diet containing 6% β-glucan [29]. Further, Christensen et al (2013) found a decreased glucose absorption in pigs fed bread enriched with AX at 60 minutes postprandial and a decreased insulin secretion at 30 minutes postprandial [9]. Other studies using wheat AX concentrates have also shown plasma glucose to be attenuated following a meal challenge [30–32].

Following an OGTT and feeding, plasma glucagon concentrations are expected to decrease as plasma insulin concentrations increase [25]. Directly after the pigs received the feed challenge, plasma glucagon concentrations remained constant and even increased. This has been previously observed in subjects with type 1 diabetes where infused insulin did not suppress α-cell production of glucagon [33, 34], but also in subjects that had tight glycaemic control [35]. Moreover, the feed contained protein in addition to carbohydrates. It is well known that a complex meal stimulates glucagon as well as insulin. Diets with protein (amino acids) stimulate both insulin and glucagon directly at the pancreatic β and α cells respectively. In doing so, they counter-balance each other in terms of glucose homeostasis. In response, co-stimulation of glucagon and insulin by amino acids was achieved, thereby offsetting any change in plasma glucose.

### Pigs fed a combination of SDF (i.e. AXBG) had significantly decreased postprandial plasma GIP concentrations, but no change in GLP-1, PYY and ghrelin responses

The release of enteroendocrine hormones is a response to nutrient signalling in the gastrointestinal tract (GIT) via intestinal receptors that respond to mechanical and/or chemical stimulation. For example, the anorexigenic hormone GLP-1 is secreted by enteroendocrine type L-cells located in the distal small intestine and the large intestine. GLP-1 is known to inhibit acid secretion and gastric emptying. Fasting plasma GLP-1 concentrations are around 8–15 pmol/L and rise to 20–35 pmol/L post-prandial [29, 36]. The rise of plasma GLP-1 is higher in response to intraduodenal infusion of fat than of glucose, but is additive when both nutrients are infused together [37]. The enteroendocrine hormone GIP is synthesized within and released from intestinal K-cells, the majority of which are located in the duodenum and proximal jejunum. GIP secretion is stimulated in response to the rate nutrient absorption and is especially sensitive to glucose or fat [38].

In response to increased plasma glucose concentrations following the feed challenge, plasma insulin concentrations increased dramatically (Fig 2). Plasma incretin concentrations (Fig 5) support the postprandial insulin surge (Fig 2B). Due to this insulin response, NEFA concentrations decreased (Fig 4) and following the feed challenge, the metabolic parameters analysed in this study commenced their return to resting states. Pigs fed the AXBG diet had significantly lower plasma GIP concentrations when compared with pigs fed the WS diet. Postprandial plasma insulin concentrations were not significantly different. Lower postprandial GIP concentrations for pigs fed diets containing AX and BG is in agreement with previous studies [9, 29]. Plasma GLP-1 concentrations were not significantly affected by the diets varying in SDF; however this is also in agreement with previous studies [9]. We hypothesize that the difference in the incretin response is due to their anatomical location and the corresponding rate of nutrient absorption that would be occurring in the respective sections. As mentioned earlier, K-type (GIP) and L-type (GLP-1) cells are predominantly located in the proximal and distal small intestine respectively. A reduced postprandial GIP response for pigs fed the AXBG diet may reflect a reduced rate of nutrient absorption in the proximal duodenum. Furthermore, accumulated evidence suggests that GIP, through its specific receptor, plays an important role in the onset of obesity by promoting energy storage in adipose tissues [39, 40]. Long-term reduced secretions of GIP may result in a reduction in adiposity. Twelve months after Roux-en-Y gastric bypass (RYGB) reversal in 25 kg pigs, GIP levels are lower and respond slower to a mixed-meal test compared to before RYGB reversal [41]. The opposite (i.e. increased GIP levels) was later shown by the same group [42] and GIP levels in humans have been shown to not be influenced by a RYGB [43].

It has been shown in previous experiments that porcine plasma PYY concentrations are higher in *ad libitum* fed pigs than fasted pigs (500 vs 200 pmol/L, respectively) [44]. However, Souza et al. showed no response of plasma PYY concentrations on a mixed-nutrient meal [36].

Fasting plasma PYY concentrations increased prior to the pigs being fed in the current study. Secretion of PYY is known to occur in anticipation of feeding, before nutrients reach the PYY-releasing cells [38]. Following the feed challenge, plasma PYY concentrations increased in response to nutrient ingestion. In response to feeding, plasma PYY concentrations peaked at 30 minutes for WS pigs, but peaked at 60 minutes postprandial for AXBG pigs. This shift in the postprandial peak of plasma PYY concentrations has been observed previously when soluble psyllium-fibre enriched meals induced a slower release of PYY [38]. A previous study using overweight but non-diabetic humans reported that total levels of plasma PYY increased in a linear fashion with increasing concentrations of soluble β-glucan [45]. There is extensive literature on the effects of ghrelin and leptin interactions in humans [46]. Despite not finding any dietary effects on ghrelin concentration due to SDF in these pigs, it has been reported that healthy humans had an increase in serum ghrelin levels after ingesting AX [47]. However, there is still debate as to whether changes in ghrelin responses are associated with acute satiety effects or with the inclusion of fibre [48].

### Fibre inclusion may account for differences observed in the incretin response due to reduced nutrient absorption occurring in the proximal duodenum

These results demonstrate that the male grower pigs studied had extremely tight glycaemic control and glucose tolerance. A biphasic response following an OGTT is therefore not exclusive to female humans and pigs [23]. Although cereal SDFs have been shown in previous studies to alter carbohydrate digestion and attenuate subsequent glycaemic responses, the current study shows that the glycaemic response can be delayed in addition to being reduced, probably mediated by altered physico-chemical properties of the diet and the physiological effects this has *in vivo*. Additionally, the current study revealed that the incretin response differed due to dietary inclusion of SDF and difference of *in situ* location of the enteroendocrine cells in the small intestine.

## Conclusions

The current study provides insights into the effects of cereal SDF on glycaemia, suggesting that there may be subtle differences depending on the type, amount and processing which may influence feed/food structure and the rate of absorption in the proximal small intestine. Further exploration of these factors, especially postprandial enteroendocrine secretions will be useful in improving our knowledge of how different SDF combinations may influence glycaemic responses and is deserving of further study.

## Supporting information

S1 File Insulin and glucose AUC for the feed challenge and the OGTT. (CSV)

S2 File Incretin, glucagon, ghrelin and PYY data for pigs. (XLSX)
